# Conductor–Insulator Interfaces in Solid Electrolytes: A Design Strategy to Enhance Li-Ion Dynamics in Nanoconfined LiBH_4_/Al_2_O_3_

**DOI:** 10.1021/acs.jpcc.1c03789

**Published:** 2021-07-06

**Authors:** Roman Zettl, Katharina Hogrefe, Bernhard Gadermaier, Ilie Hanzu, Peter Ngene, Petra E. de Jongh, H. Martin R. Wilkening

**Affiliations:** † Institute for Chemistry and Technology of Materials, Christian-Doppler-Laboratory for Lithium Batteries, Graz University of Technology (NAWI Graz), Stremayrgasse 9, 8010 Graz, Austria; ‡ Materials Chemistry and Catalysis, Debye Institute for Nanomaterials Science, Utrecht University, 3584 Utrecht, Netherlands

## Abstract

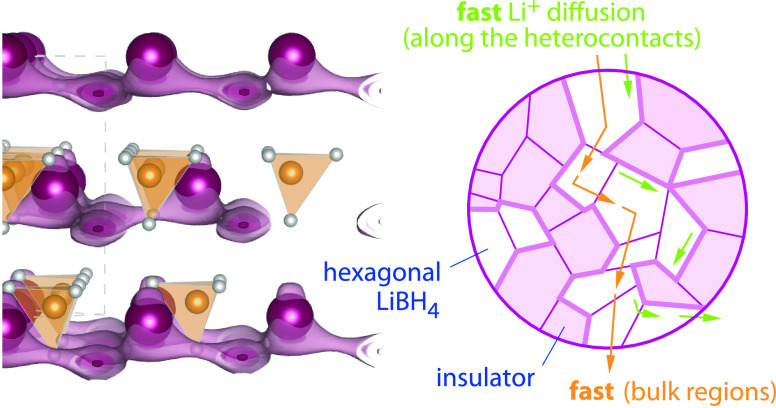

Synthesizing Li-ion-conducting solid electrolytes with application-relevant properties for new energy storage devices is a challenging task that relies on a few design principles to tune ionic conductivity. When starting with originally poor ionic compounds, in many cases, a combination of several strategies, such as doping or substitution, is needed to achieve sufficiently high ionic conductivities. For nanostructured materials, the introduction of conductor–insulator interfacial regions represents another important design strategy. Unfortunately, for most of the two-phase nanostructured ceramics studied so far, the lower limiting conductivity values needed for applications could not be reached. Here, we show that in nanoconfined LiBH_4_/Al_2_O_3_ prepared by melt infiltration, a percolating network of fast conductor–insulator Li^+^ diffusion pathways could be realized. These heterocontacts provide regions with extremely rapid ^7^Li NMR spin fluctuations giving direct evidence for very fast Li^+^ jump processes in both nanoconfined LiBH_4_/Al_2_O_3_ and LiBH_4_-LiI/Al_2_O_3_. Compared to the nanocrystalline, Al_2_O_3_-free reference system LiBH_4_-LiI, nanoconfinement leads to a strongly enhanced recovery of the ^7^Li NMR longitudinal magnetization. The fact that almost no difference is seen between LiBH_4_-LiI/Al_2_O_3_ and LiBH_4_/Al_2_O_3_ unequivocally reveals that the overall ^7^Li NMR spin-lattice relaxation rates are solely controlled by the spin fluctuations near or in the conductor–insulator interfacial regions. Thus, the conductor–insulator nanoeffect, which in the ideal case relies on a percolation network of space charge regions, is independent of the choice of the bulk crystal structure of LiBH_4_, either being orthorhombic (LiBH_4_/Al_2_O_3_) or hexagonal (LiBH_4_-LiI/Al_2_O_3_). ^7^Li (and ^1^H) NMR shows that rapid local interfacial Li-ion dynamics is corroborated by rather small activation energies on the order of only 0.1 eV. In addition, the LiI-stabilized layer-structured form of LiBH_4_ guarantees fast two-dimensional (2D) bulk ion dynamics and contributes to facilitating fast long-range ion transport.

## Introduction

Hydride-based solids attracted great attention as promising electrolytes for lithium-ion batteries^1^ due to their compatibility with Li metal and their mechanical robustness.^2,3^ While Li^+^-ion transport in polycrystalline oxide-type electrolytes^4^ may suffer from large grain-boundary resistances, such regions do not hinder long-range ion transport in the mechanically softer hydrides.^5^

**Figure 1:**
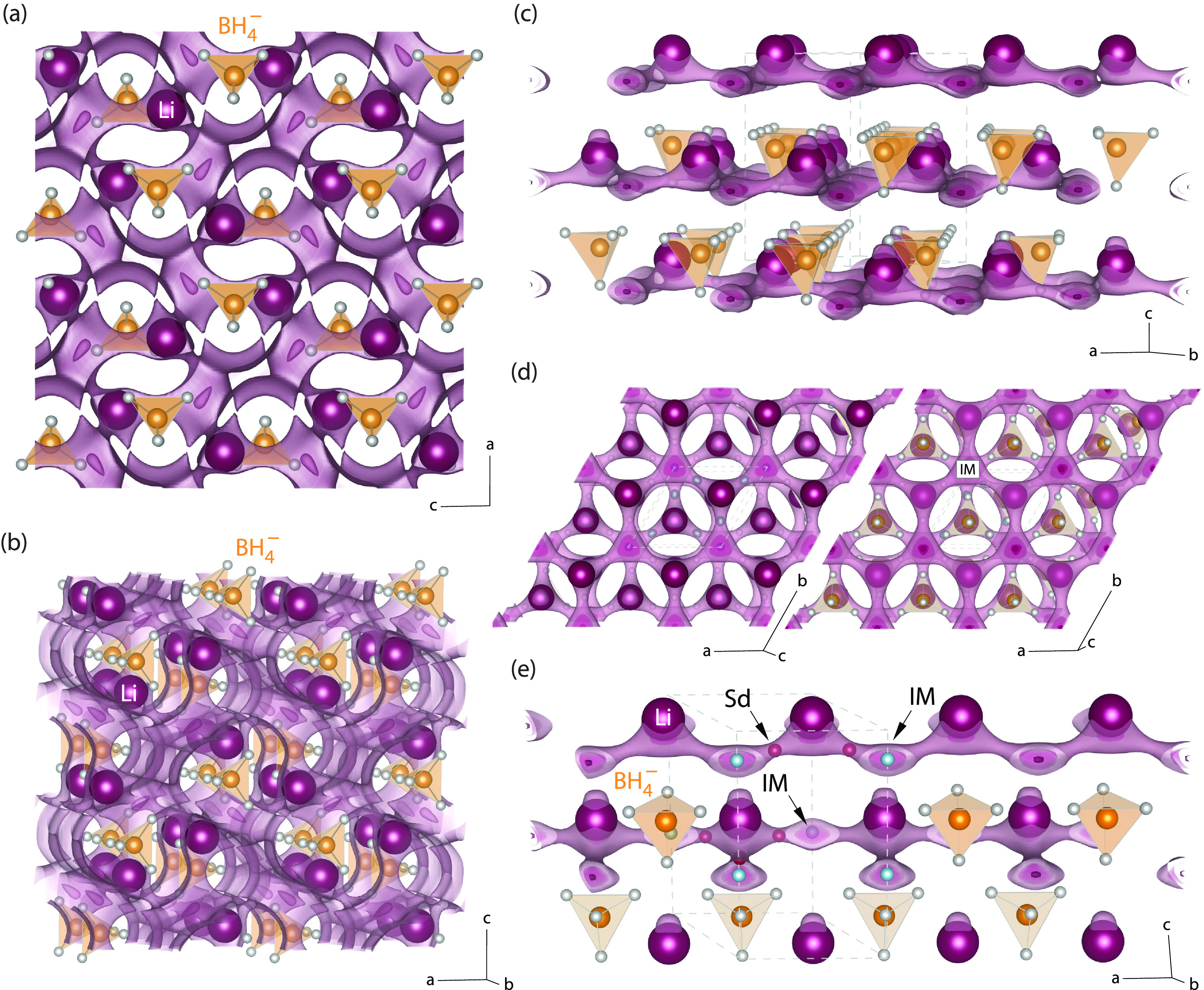
(a, b) Crystal structure of orthorhombic LiBH_4_, slightly different viewing direction as indicated by the axes drawn. The topology of possible ion-migration paths, as estimated via the SoftBV software tool and the bond valence pathway analyzer (see text), turned out to be interrupted rather than interconnected, mirroring the poor ionic conductivity of this phase. (c–e) Crystal structure of layered, hexagonal LiBH_4_ for which the Li^+^ ions preferentially diffuse in two dimensions. (d) View along the *c*-axis in both directions to visualize the next-neighbor Li^+^ jump processes on a hexagonal lattice. (e) When jumping between regularly occupied sites, the ions temporarily occupy an intermediate position (IM). The saddle points connecting IM with the regular sites are marked with Sd and represent the points of the highest energy along the migration path.

The most prominent model hydride is LiBH_4_ whose hexagonal modification, which is stable above *T*_pt_ = 110 °C, shows high conductivities in the mS cm^–1^ range.^6,7^ The corresponding orthorhombic form, being the favorable crystal structure below *T*_pt_, is, however, a rather poor ion conductor,^7^ likely because of much higher defect formation energies.^8^ While ultraslow Li^+^ ion exchange in orthorhombic LiBH_4_ is assumed to take place in three dimensions (Figure 1), for layer-structured LiBH_4_, a two-dimensional (2D) conduction mechanism prevails (Figure 1), as has been shown by both frequency-dependent ^7^Li NMR spin-lattice relaxation (SLR) measurements^9−11^ and calculations.^12^ This 2D diffusion behavior is illustrated in Figure 1 using bond valence site energy estimations.

Two approaches have been established and presented in the literature that successfully enhance the room-temperature ionic conductivity of LiBH_4_ by several orders of magnitude, viz., (i) nanoconfinement of LiBH_4_ in insulating oxides^13,14^ and (ii) and partial cationic and especially anionic substitution^6,7^ of the BH_4_^–^ units with halogen ions like I^–^, Br^–^, or Cl^–^. It is strongly anticipated that these two approaches lead to fundamentally different diffusion mechanisms. While anion substitution in LiBH_4_-LiX (X = I, Br, Cl) stabilizes the highly conductive hexagonal phase at much lower temperatures than *T*_pt_,^15^ through nanoconfinement, a large fraction of Li^+^-ion conductor–(ionic)insulator interfacial regions are introduced, which are suggested to be responsible for increased long-range ion transport. A definite proof of the latter concept or effect is, however, still missing for the LiBH_4_/Al_2_O_3_ composites. For LiBH_4_/SiO_2_ nanocomposites, the important role of surface groups has been discussed recently.^16,17^

In general, heterocontacts between two different phases, viz., an ion conductor and an insulating phase, or even between two (mixed) conductors, may generate a percolation network of space charge regions with enhanced charge carrier mobility. The most prominent two-phase system is composed of alternating layers of F^–^-ion-conducting BaF_2_ and CaF_2_ with thicknesses of 9 nm, which were grown by molecular beam epitaxy.^18^ The foundations of space charge zones in such nanostructured artificial ion conductors were laid by Maier,^19−22^ explaining such nontrivial size effects that rely on overlapping space charge zones. CuBr/Al_2_O_3_(TiO_2_) composites, as studied by Knauth and co-workers,^23^ belong to another group of such composites that show enhanced electrical conductivity.^24,25^

In the case of Li-ion conductors, Liang observed increased ionic conductivities in samples of LiI/Al_2_O_3_.^26^ Later, for the nanocrystalline system Li_2_O/X_2_O_3_ (X = B, Al), similar effects^27,28^ were observed by ^7^Li NMR SLR rate measurements.^29,30^ Although enhanced Li^+^ diffusivity was probed, the resulting conductivities^28^ could not reach any practical benchmark needed to realize all-solid-state batteries equipped with ceramic electrolytes.

This situation is, however, different for nanoconfined LiBH_4_-LiI/Al_2_O_3_ and LiBH_4_/Al_2_O_3_, both showing ionic conductivities in the order of 10^–4^ S cm^–1^,^31^ which is by more than a factor of 100 higher than in orthorhombic LiBH_4_.^13^ To understand the synthetic approaches and their impact on overall ionic transport, the role of the conductor–insulator interface in achieving such high conductivities needs to be studied in detail. Preferably, such studies should include spectroscopic methods^32^ being sensitive to local Li^+^ hopping processes in or near these interfacial areas. Here, we used ^7^Li NMR spectroscopy to quantify the effect of the conductor–insulator interfacial regions in nanoconfined LiBH_4_-LiI/Al_2_O_3_ and LiBH_4_/Al_2_O_3_ prepared by melt infiltration. Although a recent ^27^Al NMR study in our labs suggested that (unsaturated) penta-coordinated Al centers near the Al_2_O_3_ surface regions are involved in creating a defect-rich LiBH_4_/Al_2_O_3_ interface,^33^ the ultimate proof via ^7^Li NMR SLR measurements is still missing. In the present study, we directly compared the ^7^Li (and ^1^H) NMR response of longitudinal SLR of LiBH_4_(-LiI)/Al_2_O_3_ with those of bulk LiBH_4_ and LiI-stabilized LiBH_4_. We observed a tremendous effect of the insulator Al_2_O_3_ on ^7^Li NMR SLR, which is directly proportional to the diffusive motions of the Li^+^ ions, clearly showing the superior role of conductor–insulator regions in solid electrolytes with nanometer-sized dimensions. Only in such samples, the volume fraction of these regions is large enough to have a dominant effect on overall ion transport properties.

### Methods and Characterization

The composite electrolytes investigated here, i.e., LiBH_4_/Al_2_O_3_ and LiBH_4_-LiI/Al_2_O_3_, were prepared via melt infiltration; LiBH_4_-LiI served as a reference compound. A detailed description of the corresponding procedure^34^ as well as of the preparation and characterization of the composites^31^ can be found elsewhere as the same samples were used for earlier studies. Ionic substitution was realized in a molar ratio of 80:20 (LiBH_4_/LiI). The samples were kept at 295 °C for 30 min under 50 bar H_2_ pressure in a stainless steel high-pressure autoclave (Parr). The average diameter of the pores in Al_2_O_3_ is in the order of 10 nm.^31^ As mentioned above, at room temperature, bulk LiBH_4_ crystallizes with orthorhombic structure and transforms into its hexagonal phase at temperatures higher than 110 °C; the corresponding X-ray diffraction patterns are shown in Figure S1. Here, results from differential scanning calorimetry (DSC), see Figure S2, reveal that the corresponding signal of LiBH_4_/Al_2_O_3_ splits into two peaks at 103 °C and 114 °C; the signals are significantly decreased compared to the expected one of bulk LiBH_4_, which was found at 117 °C. We do not observe any diagnostic DSC signals pointing to a phase change in LiBH_4_-LiI/Al_2_O_3_ as for LiBH_4_-LiI, the hexagonal modification is stabilized by the introduction of LiI already at lower temperatures. For the sake of completeness, LiBH_4_-LiI shows a slight endothermic signal at −19 °C. The thermal behavior of LiBH_4_/Al_2_O_3_ is useful when interpreting the diffusion-induced ^7^Li NMR data, which were collected as follows.

Variable-temperature ^7^Li (and ^1^H) NMR 1/*T*_1_ SLR rates were measured with a Bruker Avance III 300 spectrometer that is connected to a 7-Tesla cryomagnet. The corresponding Larmor frequencies were 116 MHz for ^7^Li and 300 MHz for ^1^H. All samples were smoothly hand-pressed in Duran tubes under protective atmosphere and sealed. Relaxation rates were determined at temperatures ranging from −100 to 200 °C with an increment of usually 20 °C. In the region of the diffusion-induced rate peaks, additional ^7^Li NMR 1/*T*_1_ rates were recorded every 10 or 5 °C. The laboratory-frame ^7^Li (and ^1^H) NMR 1/*T*_1_ rates were acquired with the well-known saturation recovery pulse sequence; depending on temperature, the 90° pulse lengths (200 W) varied from 2.5 to 2.9 μs (^7^Li) and from 1.1 to 3.3 μs (^1^H). Usually four to eight scans were accumulated to obtain a single free induction decay. For a detailed description of the pulse programs used and for a discussion of the procedure employed to parameterize the longitudinal NMR transients (partly displayed in Figure S3), we refer to our previous study.^33^

We also performed bond valence site energy estimations combined with a bond valence pathway analyzer using the softBV software tool developed by Adams and co-workers.^35,36^ We took the structural information from published synchrotron X-ray powder diffraction data.^37^ The softBV software executes a structure plausibility check, calculates surface energies, and gives information about the positions of interstitial sites and saddle points, as well as the topology and dimensionality of ion-migration paths and the respective migration barriers.^35,36^ For the calculations, Li^+^ was chosen as the mobile ion and the grid resolution was set to 0.1 Å. Pros and cons of the approach via softBV are discussed elsewhere.^36^ For the visualization of the data, we used the VESTA software package.^38^

## Results and Discussion

### NMR Spin-Lattice Relaxation: Li-Ion Translational Dynamics and Rotational Jumps of the Polyanions

**Figure 2:**
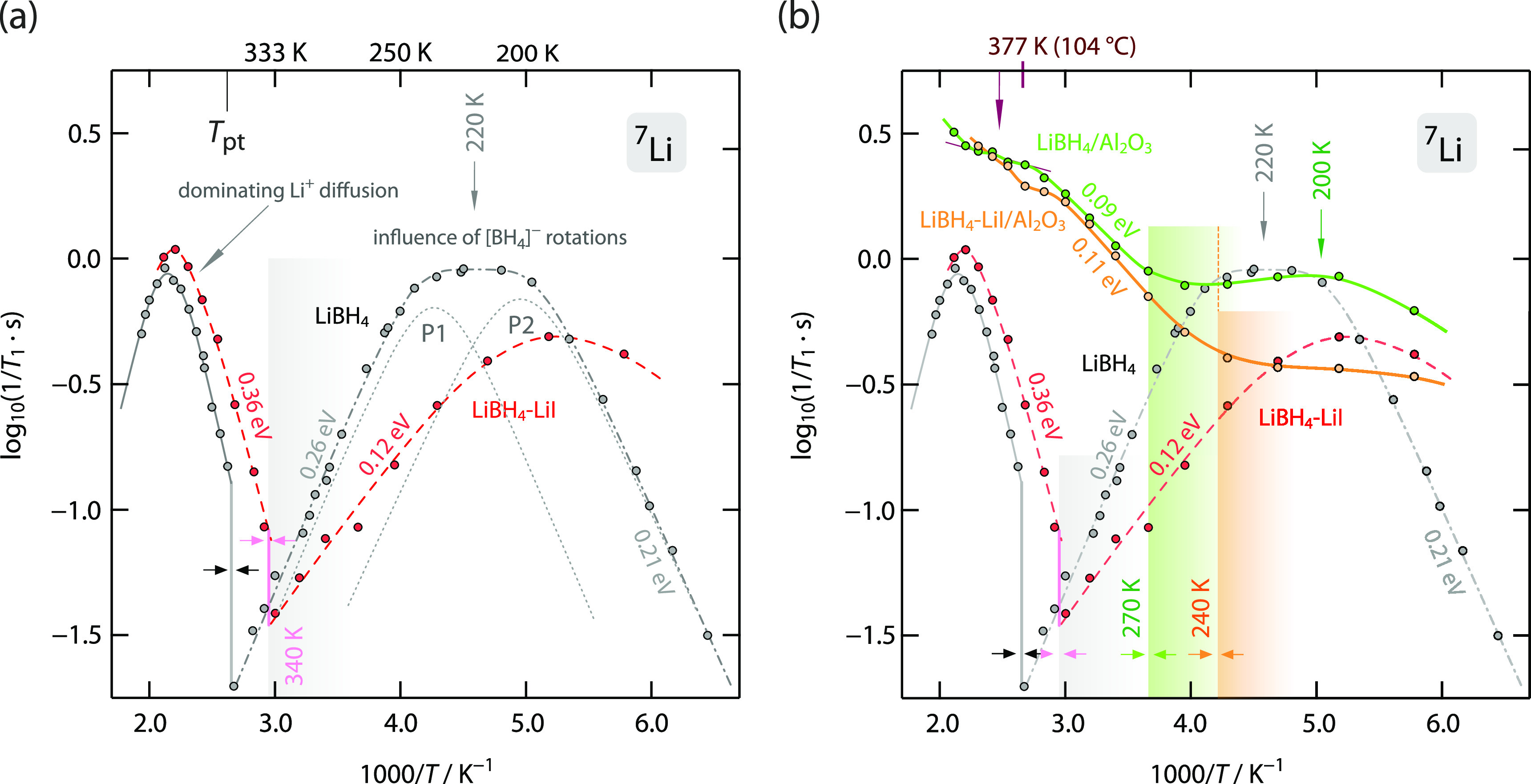
(a) Arrhenius representation of the ^7^Li NMR spin-lattice relaxation rates 1/*T*_1_ (116 MHz) of microcrystalline LiBH_4_ and the LiI-stabilized form LiBH_4_-LiI; the former rates were taken from an earlier study by some of us.^9^ Below the transition temperature of ca. 110 °C (LiBH_4_, see gray area), the rates are governed by fast rotational BH_4_^–^ dynamics in the orthorhombic form of LiBH_4_. In hexagonal LiBH_4_, 1/*T*_1_ is determined by Li^+^ translational dynamics. For LiBH_4_-LiI, the transformation temperature is reduced (see also (b)); furthermore, rotational dynamics gets enhanced as the ^7^Li NMR rate peak is shifted toward lower temperatures, it appears at ca. 190 K. Solid and dashed lines are to guide the eye; see text for further details. (b) The same representation as in (a) but with the ^7^Li NMR spin-lattice relaxation rates 1/*T*_1_ of the two nanoconfined samples included, viz., LiBH_4_-LiI/Al_2_O_3_ and LiBH_4_/Al_2_O_3_. Importantly, even for the sample free of any LiI, rather rapid Li^+^ exchange processes are probed. This observation reveals the importance of the conductor–insulator interfacial regions determining overall ^7^Li spin fluctuations in nanoconfined LiBH_4_/Al_2_O_3_. Activation energies refer to the almost linear regions of the 1/*T*_1_(1/*T*) dependence. Values as low as 0.1 eV point to an extremely flat potential landscape characterizing the conductor–insulator heterocontacts. See text for further explanation.

As mentioned above, LiBH_4_ crystallizes either with orthorhombic or with hexagonal symmetry. At temperatures lower than *T*_pt_ = 110 °C, the poorly conducting orthorhombic modification is present (see Figure 1). In ortho-LiBH_4_, the ^7^Li NMR spin-lattice relaxation rates indirectly sense the rapid rotational BH_4_^–^ dynamics rather than Li^+^ translational diffusion (see Figure 2a). Thus, at temperatures below *T*_pt_, the ^7^Li NMR rates pass through two rate peaks that mirror the two distinct rotational jump processes of the BH_4_^–^ polyanions (see Figure 2a), which shows the ^7^Li NMR SLR rates of coarse-grained, that is, microcrystalline LiBH_4_. The broad rate maximum located at 220 K is composed of two individual rate peaks, which are represented by dotted lines and labeled P1 and P2 in Figure 2a. These rate peaks were analyzed in detail by both NMR^9,39,40^ and quasi-elastic and inelastic neutron scattering earlier;^41^ additionally, the mobility of boron atoms is discussed elsewhere.^42^

Above *T*_pt_, the overall ^7^Li NMR response in bulk LiBH_4_ is governed by rapid Li^+^ (translational) jump processes in the layer-structured form of LiBH_4_. This dynamic process, which is 2D in nature as is illustrated in Figure 1, produces a single rate peak that points to an activation energy of 0.5 eV. In general, diffusion-induced ^7^Li NMR rate peaks appear if the motional correlation rate 1/*τ*_c_, which is expected within a factor of ca. 2 to be identical with the jump rate 1/τ, reaches the order of the (angular) Larmor frequency ω_0_.^11^ At the temperature where the peak appears, the condition ω_0_τ ≈ 1 is fulfilled.^43^ A symmetric rate peak is only obtained for uncorrelated and isotropic (three-dimensional, 3D) diffusion. In many cases, structural disorder combined with Coulomb interactions results in asymmetric NMR peaks whose low-*T* flank shows a lower slope than that characterizing the flank on the high-*T* side.^43^ Moreover, while the slope in the high-*T* regime is characteristic for long-range ion diffusion, the low-*T* flank of the peak is sensitive to short range, that is, local diffusion processes.^11^

Stabilizing the hexagonal phase of LiBH_4_ by the incorporation of LiI leads to several changes of the overall ^7^Li NMR response (see Figure 2a). First, it shifts the phase transition toward lower temperatures. Consequently, the ^7^Li NMR 1/*T*_1_(1/*T*) rates pass into the low-*T* flank of the rate peak, which characterizes translational Li^+^, dynamics already at temperatures equal to or larger than 340 K. In agreement with faster Li^+^ diffusion in LiBH_4_-LiI, the slope of the low-temperature flank of the rate peak yields an activation energy *E*_a_ of 0.36 eV (Figure 2a) instead of 0.5 eV for LiBH_4_.^9^ This comparison shows that LiI does not only stabilize the hexagonal form at lower *T* but also reduces the mean activation barrier for Li^+^ translational diffusion as it is seen by NMR (Figure 2a).

Apart from the change of the rates above *T*_pt_, we recognize that also the rotational jump processes change when going from microcrystalline LiBH_4_ to nanocrystalline LiBH_4_-LiI. The original, overall maximum located at 220 K shifted toward a much lower temperature (Figure 2a), indicating an increase in the corresponding motional correlation rate sensed by the ^7^Li spins. We assume that this increase is a direct consequence of the expanded lattice through the incorporation of I^–^ having a larger radius than BH_4_^–^. A deconvolution of this response into two rate peaks turned out to be no longer possible; for the LiI-containing sample LiBH_4_-LiI, the former two rate peaks P1 and P2 (see above) merge into a much broader peak located at *T* ≈ 190 K. Most likely, this change originates from a broader distribution of rotational jump rates in LiBH_4_-LiI. The shift toward lower temperatures agrees with a reduction of the activation energies *E*_a_ associated with the BH_4_^–^ rotational jumps. Here, *E*_a_ decreases from 0.26 to 0.12 eV if we consider the high-*T* flank of the ^7^Li NMR rate peaks just below *T*_pt_ (see Figure 2a). The introduction of LiI does also reduce the overall NMR coupling constant determining the maximum rates at *T* = 190 K.

Figure 2b shows the NMR responses of the two nanoconfined samples, LiBH_4_-LiI/Al_2_O_3_ and LiBH_4_/Al_2_O_3_. Starting from low temperatures, we recognize that the rates evolve in a similar manner to those of LiBH_4_ and LiBH_4_-LiI, respectively. However, especially for LiBH_4_/Al_2_O_3_, without any LiI incorporated, we notice enhanced BH_4_^–^ rotational dynamics compared to the microcrystalline reference sample LiBH_4_ having no contact to any insulator phase. For LiBH_4_-LiI/Al_2_O_3_ and LiBH_4_-LiI, the ^7^Li response turned out to be rather similar, while variable-temperature ^1^H NMR SLR measurements (Figure S4) showed some subtle differences in this temperature regime (see the Supporting Information). As suggested by Figures S4 and 2b, rotational ion dynamics in or near the conductor–insulator interfacial regions are enhanced for LiBH_4_(-LiI)/Al_2_O_3_ compared to those in the bulk regions of LiBH_4_.

Most importantly, the largest effect of the insulating phase on ^7^Li NMR spin-lattice relaxation is seen at higher temperatures when Li^+^ translational ion dynamics start to govern the spin fluctuations (Figure 2b). In contrast to the sample without any Al_2_O_3_, we clearly recognize that the ^7^Li NMR rates start to increase at temperatures as low as 240 and 270 K, respectively. These temperatures are clearly lower than *T*_pt_ = 340 K for LiBH_4_-LiI (see Figure 2a). At temperatures slightly above 270 K, we recognize that the LiI-free sample does almost show the same NMR SLR response as seen for LiBH_4_-LiI/Al_2_O_3_, which unequivocally reveals that the interface effect is the main reason for the longitudinal recovery of the magnetization mirroring Li^+^ diffusivity.

Here, this effect turned out to be much larger than that seen for LiBH_4_/Al_2_O_3_ composites that were earlier prepared by (high-energy) ball milling.^13^ Melt infiltration leaves behind a defective LiBH_4_ phase, and we assume tight conductor–insulator contacts. The nanoconfined samples provide a large fraction of these heterocontacts. Our comparative NMR results clearly show that the interfacial regions play a dominant role in explaining enhanced ion dynamics in the nanoconfined samples regardless of whether LiI is present or not. The latter finding is supported by recent calculations revealing that the poor ion transport in orthorhombic LiBH_4_ originates from very high defect formation energies.^8^ The LiBH_4_/Al_2_O_3_ zones are, however, expected to be rich in defects, thus facilitating ion transport.^33^ A similar effect has been described very recently by first-principles calculations for the interface in LiBH_4_/MoS_2_ composites.^44^ Importantly, in LiBH_4_/SiO_2_ composites, the role of surface groups should not be underestimated.^16,17^ Surface effects are also important for LiBH_4_/Al_2_O_3_: as has been shown quite recently by ^27^Al NMR,^33^ penta-coordinated Al centers Al^IV^ get saturated while generating Al^IV^BH_4_^–^–Li^+^, forming a defect-rich zone with vacant or interstitial Li^+^ sites. The same mechanism has also been proposed by some of us for the recently studied LiF/Al_2_O_3_ nanocrystalline composites.^45^

Up to 330 K, the ^7^Li NMR rates of the two nanoconfined Al_2_O_3_-containing samples (see Figure 2b) follow linear behavior. The associated activation energies turn out to be rather low and take values in the order of only 0.1 eV. This average value mirrors a flat potential landscape and is even lower than that of nanocrystalline LiBH_4_ (0.18 eV) prepared by ball milling.^46^ Values in the order of 0.07 eV were also observed indirectly by ^1^H NMR SLR measurements above 380 K (see Figure S4, Supporting Information). Again, we ascribe the reduction in activation energy when going from nanostructured LiBH_4_ to nanoconfined LiBH_4_/Al_2_O_3_ to the interfacial “insulator effect” generating a percolation network of fast diffusion pathways for the Li^+^ ions. Most likely, as detailed above, such a network benefits from defect-rich space charge regions that influence the Li^+^ hopping processes.

The 1/*T*_1_ rates of nanoconfined LiBH_4_/Al_2_O_3_ level off near 373 K and pass into a region that is characterized by a very low activation energy (see arrow in Figure 2b). In this region, the phase transition from orthorhombic to hexagonal LiBH_4_ is marked by anisothermic peaks in our DSC measurements (see Figure S2, Supporting Information). As the addition of LiI stabilizes the hexagonal phase at temperatures well above room temperature, the anisothermic peaks are virtually absent. Again, this comparison shows that it is not the crystal structure but rather the interaction with the Al_2_O_3_ interfaces that governs ion dynamics in the interfacial regions of the nanoconfined composite samples below the phase transition temperature. Our results show how this effect can be used to turn a poor ion conductor, such as orthorhombic LiBH_4_, into a superior material with fast Li^+^ exchange processes assisting in facile macroscopic ionic transport (see below).

### ^7^Li NMR Line Shapes of the Nanoconfined Composites

**Figure 3:**
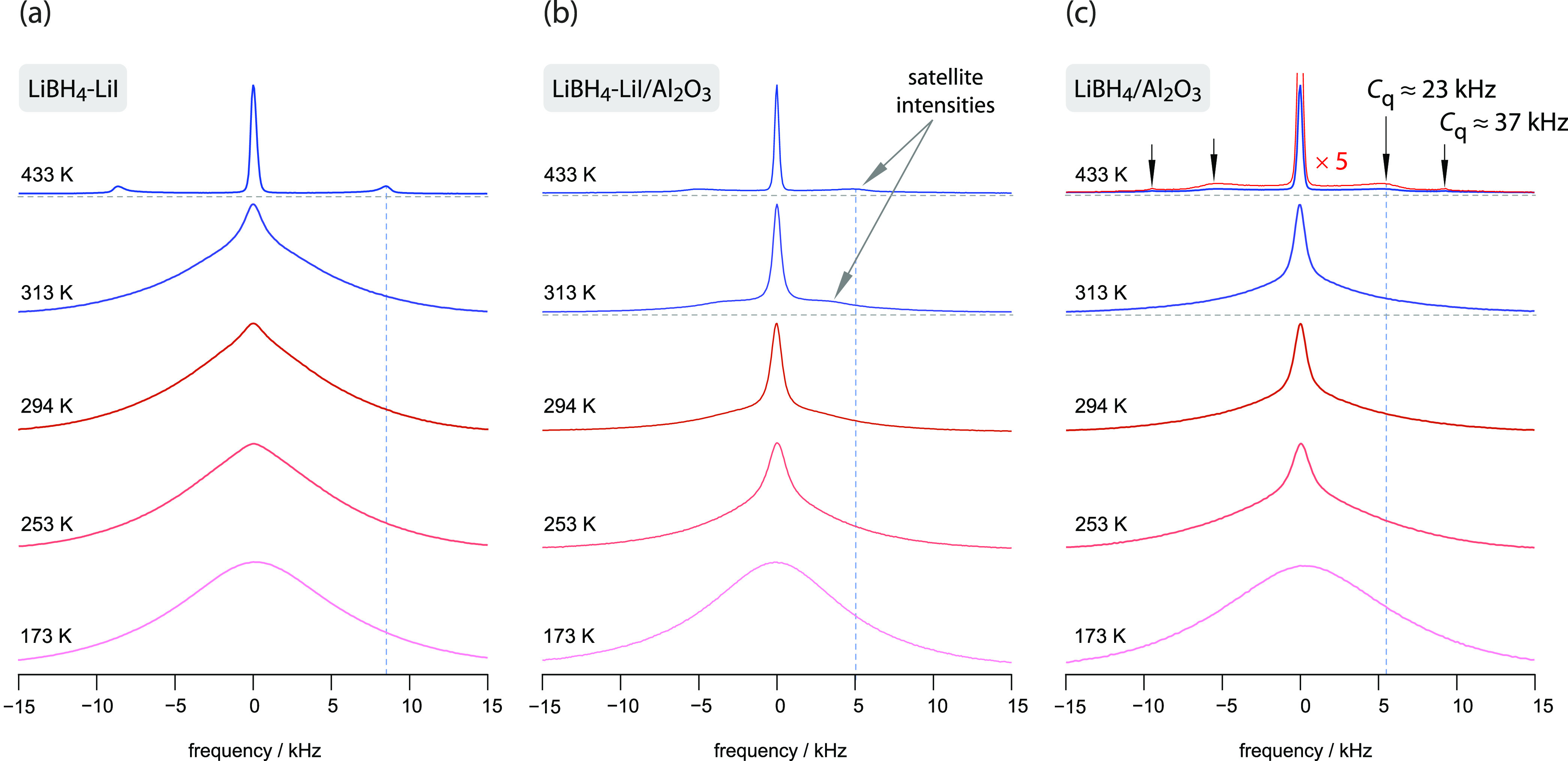
(a–c) Variable-temperature ^7^Li NMR line shapes of the three samples studied, including the reference material LiBH_4_-LiI. We observe distinct differences when the responses of the Al_2_O_3_-containing nanoconfined samples (see (b) and (c)) are compared with that of LiBH_4_-LiI (a). For LiBH_4_-LiI heterogeneous motional narrowing sets in at 294 K, stepwise narrowing is ascribed to two spin reservoirs with one of them representing the much less mobile Li^+^ ions in the bulk regions crystallizing with orthorhombic structure at very low *T*. As LiI stabilizes the hexagonal form well above room temperature, the narrow line at elevated *T* reflects Li^+^ ions in the hexagonal phase. For LiBH_4_-LiI/Al_2_O_3_ and even for LiBH_4_/Al_2_O_3_, the narrowing process is clearly shifted toward much lower temperatures of *T* < 253 K revealing that the conductor–Al_2_O_3_ interfacial regions govern overall Li^+^ translational motions sensed by the ^7^Li NMR spectra. Compared to the spectra shown in (a), a large fraction of Li ions benefits from this insulator or interface effect. The magnified spectrum in (c) shows the quadrupole powder pattern of LiBH_4_/Al_2_O_3_. Dashed (vertical) lines refer to the position of the quadrupole singularities on the kHz scale; see text for further details.

While NMR spin-lattice relaxation rates, especially when probed in the temperature regime of the low-*T* flank of the given 1/*T*_1_(1/*T*) rate peak, are sensitive to the elementary hopping processes, NMR line shapes, which are governed by spin-spin-relaxation rates, can be used to probe Li^+^ transport on longer length scales. To see whether and to which extent the conductor–insulator effect does also affect the corresponding ^7^Li NMR lines, we recorded variable-temperature spectra of the reference sample LiBH_4_-LiI (see Figure 3a) and the two nanoconfined samples (see Figure 3b,c).

Figure 3a shows the ^7^Li NMR lines of nanocrystalline LiBH_4_-LiI. Starting from a broad signal at 173 K, which reveals sluggish Li^+^ translational ion dynamics in the bulk regions, the line undergoes heterogeneous motional narrowing upon heating. At temperatures above 294 K, a narrow line superimposes the broader Gaussian-shaped main signal. We attribute the narrowed line to Li^+^ ions in the interfacial regions of this nanocrystalline sample. These regions offer fast Li^+^ diffusion pathways as has recently been shown for nanocrystalline, orthorhombic LiBH_4_.^46^ The line recorded at 433 K reflects Li^+^-ion dynamics in hexagonal LiBH_4_-LiI. At this temperature, all Li^+^ ions take part in rapid exchange processes. The quadrupolar satellite signals seen at ±8 kHz represent the 90° singularities of the powder pattern that is diagnostic for this sample. In general, quadrupole intensities mirror the interaction between the electric quadrupolar moment of the ^7^Li nucleus (spin-quantum number *I* = 3/2) with a nonvanishing electric field gradient (EFG) at the nuclear site. This interaction alters the Zeeman levels such that for *I* = 3/2, four inequivalent levels are generated that depend on the crystallite orientation in the external magnetic field. Assuming an (averaged) axially symmetric EFG at the nuclear sites, the corresponding quadrupolar coupling constant *C*_q_ of the powder sample is given by *C*_q_ = 32 kHz.

Nanoconfinement, i.e., the introduction of conductor–insulator interfacial regions, ensures that (heterogeneous) motional narrowing does already set in at temperatures lower than 250 K. This temperature agrees with the temperature at which the ^7^Li NMR rates start to increase. Satellite singularities come into the picture at 313 K. Already at 294 K, approximately 50% of the Li^+^ ions in LiBH_4_-LiI/Al_2_O_3_ (see Figure 3b) have access to fast diffusion pathways as is indicated by the ratio of the area fractions of the broad and the narrow NMR lines. For LiBH_4_/Al_2_O_3_, the area fraction of the narrow line amounts to ca. 30% at 294 K (see Figure 3c).

As discussed earlier,^31,33^ the overall coupling constant *C*_q_ (≈ 20 kHz) turned out to be clearly reduced compared to that of LiBH_4_-LiI. Importantly, for LiBH_4_/Al_2_O_3_, almost the same line shapes are detected as for the LiBH_4_-LiI/Al_2_O_3_. Again, this result demonstrates the leading role of Al_2_O_3_ in governing the ^7^Li NMR signals. Motional narrowing is slightly shifted toward higher *T*, which is in excellent agreement with the temperature behavior of the ^7^Li NMR rates. The corresponding coupling constant *C*_q_ = 23 kHz of LiBH_4_-Al_2_O_3_ resembles that of LiBH_4_-LiI/Al_2_O_3_; see also the magnified spectrum recorded at 433 K (Figure 3c). It shows that nanoconfinement is also responsible for the electric quadrupole interactions and the majority of Li spins are subjected to in or near the conductor–insulator interfacial regions. As the pore size of Al_2_O_3_ is less than 10 nm, as has been reported earlier,^31^ bulk regions, if confined to such small cages, are obviously also affected by the insulating surface regions.^31,33^ The magnification of the spectrum recorded at 433 K (see Figure 3c) shows an additional pair of satellite regions, which we earlier ascribed to the Li ions farther away from the interface regions. The corresponding coupling constant of ca. 37 kHz agrees well with that which is obtained for pure LiBH_4_ at this temperature. Hence, NMR is able to reveal the different electrical interactions the spins are sensing in nanoconfined LiBH_4_/Al_2_O_3_, with most of them being subjected to the insulator surface interactions and some residing in the smaller bulk areas.^31^ As mentioned above, this view is also corroborated by the NMR central lines shown in Figure 3.

Importantly, fast spin diffusion connecting the two spin reservoirs in the nanoconfined samples causes single exponential ^7^Li NMR *T*_1_ magnetization transients; thus, a separation of the two spin ensembles, as it was possible earlier for high-energy ball-milled LiBH_4_, is almost impossible if we use the longitudinal transients for this purpose (see Figure S2). Moreover, at a given temperature, the associated 1/*T*_1_^7^Li NMR rates of the fast and slowly decaying part of the underlying free indication decays do almost coincide. Hence, we conclude that for the nanometer-sized architecture in the conductor–insulator composites, rather efficient spin diffusion is present. Hence, from the point of view of SLR NMR, LiBH_4_ in LiBH_4_/Al_2_O_3_ appears as an almost homogeneous phase.

**Figure 4:**
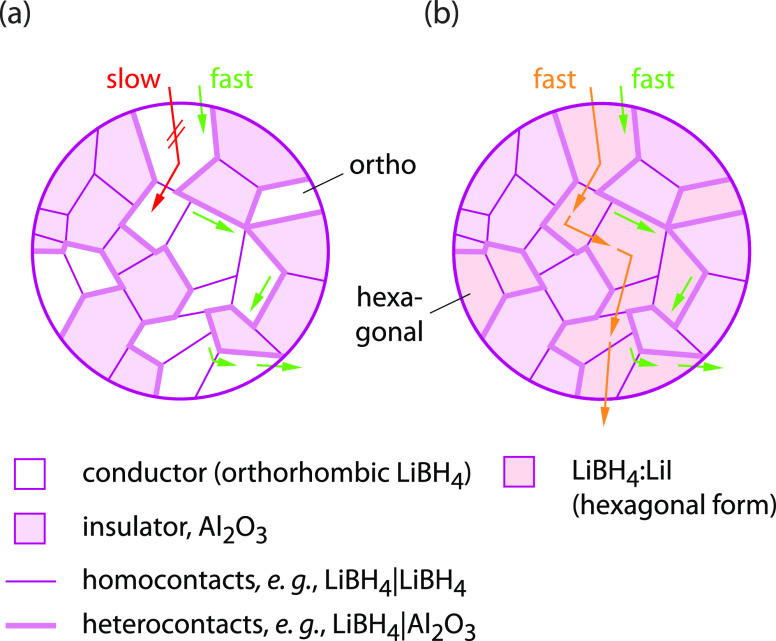
General schematic presentation of a system composed of two nanocrystalline phases, that is, an ionic conductor and a phase acting as an ionic insulator. (a) LiBH_4_/Al_2_O_3_ and (b) LiBH_4_-LiI/Al_2_O_3_ composites and the two different kinds of Li^+^ diffusion pathways present. In both cases, ^7^Li NMR is able to trace rapid fast Li^+^ self-diffusivity along (or near) the interfacial pathways generated by the conductor–insulator heterocontacts. However, if no percolation pathways are formed, the poorly conducting orthorhombic phase hinders long-range (through-going) Li^+^ ion transport (see (a)), whereas for LiBH_4_-LiI/Al_2_O_3_, fast 2D Li^+^ diffusion in the LiBH_4_-LiI bulk regions^33^ ensures facile long-range cation motions. As ionic conductivity depends on both the mobility μ and the charge carrier density *N*, the combination of anion substitution and nanoconfinement is a perfect tool to tune overall Li^+^ transport.^31,33^

Noteworthy, while NMR is able to monitor the fast Li^+^ exchange processes in the interfacial regions of the nanocomposites, it is, in the present case, less sensitive to long-range ion transport in LiBH_4_-(LiI)/Al_2_O_3_. While the two samples LiBH_4_-LiI/Al_2_O_3_ and LiBH_4_/Al_2_O_3_ show almost the same ^7^Li NMR response, through-going ionic transport in LiBH_4_-LiI/Al_2_O_3_ is easier (ca. 1.3 × 10^–4^ S cm^–1^ (298 K))^31^ than in LiBH_4_/Al_2_O_3_ (0.3 × 10^–4^ S cm^–1^ (298 K)).^31^ Most likely, this difference originates from the orthorhombic bulk regions in the latter compound that hinder Li^+^ ion dynamics; see the schematic illustration in Figure 4 that summarizes the findings. A similar picture has been proposed for other dispersed ion conductors, such as nanocrystalline Li_2_O/Al_2_O_3_ composites, whose heterogeneous transport properties were explained by the percolation concept.^47,48^ Here, for both compounds, the LiBH_4_/Al_2_O_3_ interface (heterocontacts) provides fast Li^+^ diffusion pathways. While at low temperatures Li^+^ diffusion in the orthorhombic bulk regions of LiBH_4_ in LiBH_4_/Al_2_O_3_ is slow, and only enhanced at the LiBH_4_/LiBH_4_ homocontacts, anion substitution in LiBH_4_-LiI/Al_2_O_3_ additionally ensures fast Li^+^ self-diffusivity in the hexagonal bulk regions that do not benefit from interactions with the surface regions. Therefore, the combination of nanoconfinement and anion substitution enables facile, overall Li^+^ long-range ion transport as it is necessary for, e.g., battery applications.^31,33^

## Conclusions

Using LiBH_4_ and nanoconfined LiBH_4_-LiI as model systems, we investigated the influence of conductor–insulator interfacial regions on the overall Li^+^ translational ion dynamics, which we sensed by ^7^Li NMR spin fluctuations. Al_2_O_3_ served as an insulating phase, that is, the phase usually blocking Li^+^-ion transport. As irregular diffusive motions trigger (longitudinal) ^7^Li NMR spin-lattice relaxation, with the help of variable-temperature measurements, activation energies and motional correlation rates can be probed.

While in nanoconfined LiBH_4_-LiI rapid Li^+^ translational motions (0.36 eV) influence the ^7^Li NMR rates 1/*T*_1_ at temperatures above 340 K, in the Al_2_O_3_-bearing nanoconfined samples, a drastic change in overall NMR response is seen. For LiBH_4_-LiI/Al_2_O_3_, the low-*T* flank of the corresponding diffusion-induced rate peak 1/*T*_1_(1/*T*) is already seen at a temperature as low as 240 K, thus shifted by 100 K toward lower temperatures. Surprisingly, above 270 K, the same flank is also seen for the LiI-free sample unequivocally showing that the conductor–insulator (Al_2_O_3_) effect has the authoritative role to explain the enhanced Li^+^-ion dynamics in these samples. Clearly, reduced activation energies in the order of only 0.1 eV agree with the low onset temperatures and underpin the idea of a percolation network of fast diffusion pathways generated by the conductor–insulator interfacial regions.

^7^Li NMR line shape measurements corroborate the results from ^7^Li NMR relaxometry and reveal, for both samples LiBH_4_-LiI/Al_2_O_3_ and LiBH_4_/Al_2_O_3_, an ensemble of mobile Li^+^ ions being subjected to rapid diffusive motions already at temperatures well below ambient. ^7^Li NMR quadrupole interactions seen in the spectra of nanoconfined LiBH_4_/Al_2_O_3_ do reflect both bulk and interfacial regions, with the spins in the latter areas being highly mobile and benefiting from the interaction with the insulator surface. Our work highlights the importance of conductor–insulator interfacial regions in advanced solid electrolyte research. The clever introduction of such artificial interfaces, influencing ion dynamics by both structural disorder and space charge regions, represents an adjustable tool to manipulate overall (Li^+^)ion dynamics in solids with nanometer-sized dimensions.

## Supporting Information Available

^7^Li and ^1^H NMR magnetization transients, ^1^H NMR rates, DSC curves, and XRD patterns (PDF)
