# Supplementary material for: Conductor–Insulator Interfaces in Solid Electrolytes: A Design Strategy to Enhance Li-Ion Dynamics in Nanoconfined LiBH4/Al2O3
Source: J Phys Chem C Nanomater Interfaces. 2021 Jul 6;125(27):15052–60. doi: 10.1021/acs.jpcc.1c03789 (PMC8287566; doi:10.1021/acs.jpcc.1c03789)
Supplement: Supplementary file 1 — jp1c03789_si_001.pdf [file jp1c03789_si_001.pdf]

Supporting Information

Conductor:Insulator Interfaces in Solid Electrolytes: A Design Strategy to Enhance Li-Ion Dynamics in Nanoconfined $LiBH_4$-LiI:Al_2O_3

Roman Zettl,[1,2] Katharina Hogrefe,[1] Bernhard Gadermaier,[1] Ilie Hanzu,[1] Peter Ngene,[2] Petra E. de Jongh,[2] and H. Martin R. Wilkening[1]*

[1] *Institute for Chemistry and Technology of Materials, Christian-Doppler-Laboratory for Lithium Batteries, Graz University of Technology (NAWI Graz), Stremayrgasse 9, 8010 Graz, Austria*

[2] *Materials Chemistry and Catalysis, Debye Institute for Nanomaterials Science, Utrecht University, Utrecht 3584, Netherlands*

*e-mail: wilkening@tugraz.at

X-Ray Diffraction

X-ray diffraction was performed with a Bruker-AXS D-8 Advance X-ray diffractometer with Co $K_{\alpha\,1,2}$ radiation (λ =1.79026 Å). The samples were placed in an airtight sample holder, and diffractograms were recorded at room temperature covering a 2θ range of 10° to 100°. At this temperature, $LiBH_4$ in $LiBH_4/Al_2O_3$ adopts the orthorhombic structure. For the $LiBH_4$-LiI samples we that broadening of the reflexes is due to nm-sized crystallites. Measurement details, see elsewhere.[1]

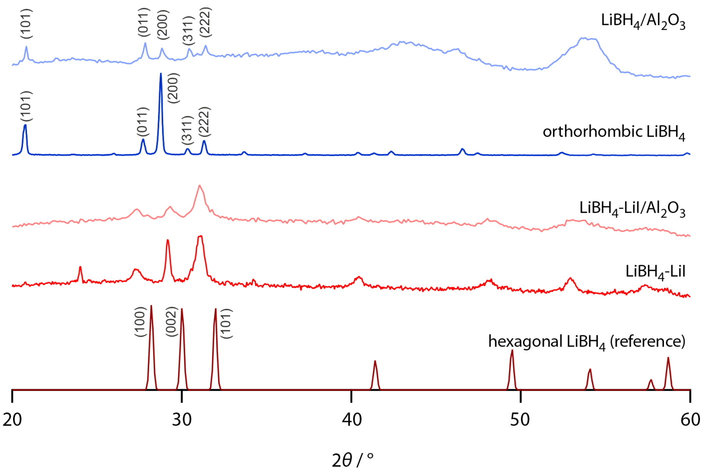

Figure S1. X-ray powder patterns of the three samples investigated. Even for $LiBH_4$-LiI we see that broadening of the reflexes points to a sample with nm-sized dimensions of the crystallites. Even smaller crystallites (< 10 nm) characterize the nanoconfined sample $LiBH_4$-LiI. For $LiBH_4/Al_2O_3$ we recognize the typical pattern of the orthorhombic phase.

Differential Scanning Calorimetry (DSC)

DSC measurements of $LiBH_4$-LiI and $LiBH_4/Al_2O_3$ were performed on a Perkin Elmer DSC 8500. For comparison, DSC curves of $LiBH_4$ and the combined composite $LiBH_4$-LiI/Al_2O_3 were recorded as well. The temperature range

of the measurement was set from −60 °C to 100 °C with a rate of 5 °C/min. The measurement program is listed below and the second heating cycle (step 7) was used to compare the samples (see Figure S1).

1) Temperature is set to 30 °C
2) Cooling from 30 °C to −60 °C
3) Holding for 3 min at −60 °C
4) Heating from −60 °C to 100 °C at a rate of 5 °C/min
5) Cooling from 100 °C down to −60°C at a rate of 5 °C/min
6) Holding for 3 min at −60 °C
7) Heating form −60 °C to 100 °C at a rate of 5 °C/min

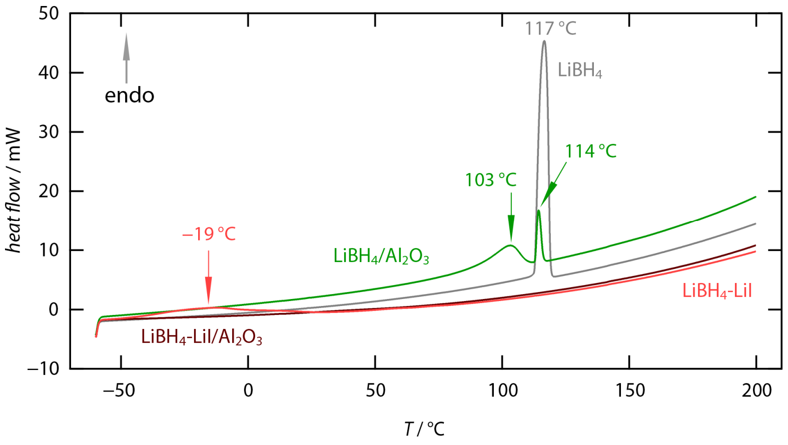

Figure S2. Dynamic scanning calorimetry (DSC) results obtained for the compounds $LiBH_4$-LiI/Al_2O_3, $LiBH_4$/Al_2O_3, $LiBH_4$-LiI, and $LiBH_4$.

T_1 NMR transients of ^{7}Li and ^{1}H SLR NMR

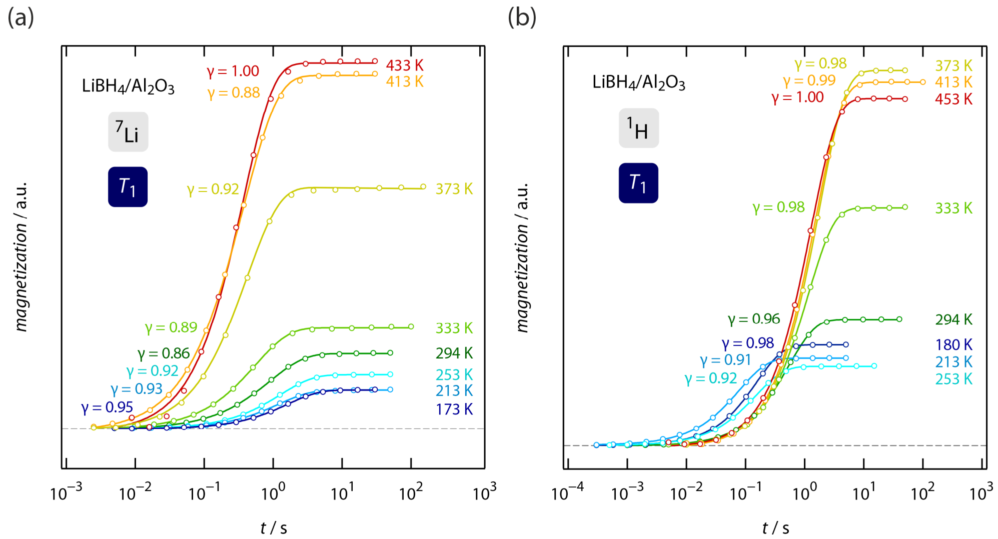

Figure S3. Selected ^{7}Li and ^{1}H NMR transients describing longitudinal T_1 spin-lattice relaxation (SLR) in the $LiBH_4$/Al_2O_3 nanocomposites: (a)^{7}Li NMR T_1 SLR transients of $LiBH_4$/Al_2O_3, (b) ^{1}H NMR SLR T_1 transients of $LiBH_4$/Al_2O_3. Solid lines show fits with stretched exponentials, the stretching factor γ is indicated.[2] The transients show almost single exponential behavior.

^{1}H NMR spin-lattice relaxation rates

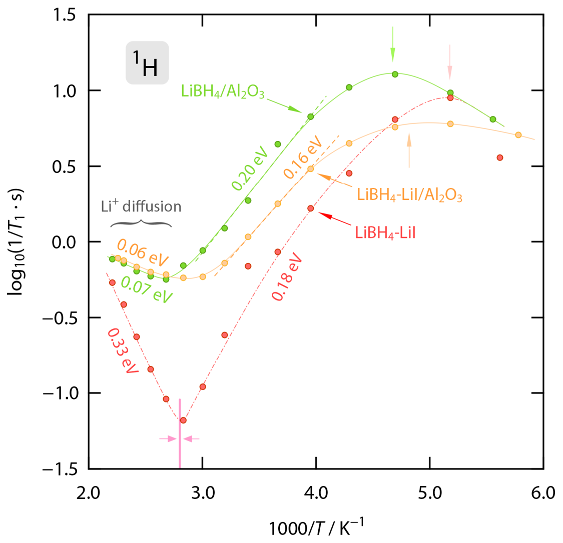

Figure S4. ^{1}H NMR SLR rates (300 MHz) as obtained for the samples indicated; the purely diffusion-induced rates $1/T_1$ were recorded in the laboratory frame with the saturation recovery pulse sequence. ^{1}H NMR shows that the re-orientational motions of the BH_4^- units are somewhat faster in $LiBH_4$ stabilized with LiI as the corresponding maximum appears at lower T. Interestingly, above 380 K the rates for the nanoconfined samples coincide and are determined by Li^+ translational diffusion and point to rather low activation energies.

References

1. Zettl, R.; de Kort, L.; Gombotz, M.; Wilkening, H. M. R.; de Jongh, P. E.; Ngene, P., Combined Effects of Anion Substitution and Nanoconfinement on the Ionic Conductivity of Li-Based Complex Hydrides. *J. Phys. Chem. C* **2020,** *124* (5), 2806-2816.

2. Zettl, R.; Gombotz, M.; Clarkson, D.; Greenbaum, S. G.; Ngene, P.; de Jongh, P. E.; Wilkening, H. M. R., Li-Ion Diffusion in Nanoconfined $LiBH_4$-LiI/Al_2O_3: From 2D Bulk Transport to 3D Long-Range Interfacial Dynamics. *ACS Appl. Mater. Interfaces* **2020,** *12* (34), 38570-38583.
